# Degradation and hydrate phase equilibria measurement methods of monoethylene glycol

**DOI:** 10.1016/j.mex.2018.12.004

**Published:** 2018-12-04

**Authors:** Khalid Alef, Stefan Iglauer, Ahmed Barifcani

**Affiliations:** a WA School of Mines: Minerals, Energy and Chemical Engineering, Curtin University, Bentley, W.A., 6102, Australia; b School of Engineering, Petroleum Engineering Discipline, Edith Cowan University, Joondalup, W.A., 6027, Australia

**Keywords:** Degradation and hydrate phase equilibria measurement methods of monoethylene glycol, Gas hydrates, Mono ethylene glycol, High pressure, PVT cell, Degradation, Reclamation

## Abstract

Monoethylene glycol (MEG), a common chemical used for the inhibition of gas hydrate formation may undergo degradation in the regeneration/reclamation process. Limited research exists on the effect of degradation of MEG on hydrate formation, production facilities and equipment especially in the presence of other chemical additives. The proposed method allows for streamlining the process of preparing, degrading and analysis of MEG solutions for hydrate testing and degradation products.

• Procedure to prepare accurate MEG solutions avoiding oxidative degradation of MEG (i.e., controlling oxygen ingress).
• Two methods are suggested to mimic field-like degradation of MEG solutions (i.e., degradation by reclamation and autoclave).
• Adoption of the isochoric hydrate testing method while using a high pressure cell with the aid of a computer script to accurately evaluate hydrate phase equilibria conditions.

## Graphical abstract

**Figure:**
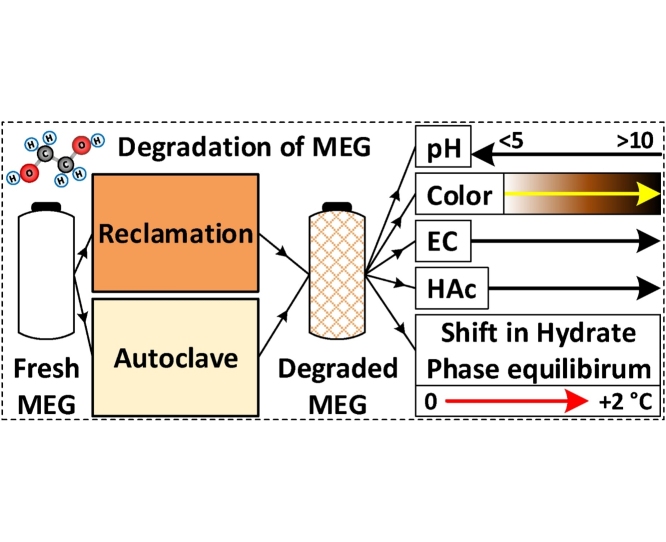

**Specifications Table**

**Table:** 

**Subject Area**	*Engineering*
**More specific subject area:**	*Hydrate Phase Equilibria*
**Method name:**	*Degradation and hydrate phase equilibria measurement methods of monoethylene glycol*
**Name and reference of original method**	
**Resource availability**	

## Method details

To meet energy demands, Natural gas has increasingly become a profitable alternative. However, a serious challenge is the formation of gas hydrates. The traditional technique to inhibit hydrate formation in pipelines is the injection of a thermodynamic hydrate inhibitor to shift the hydrate phase equilibrium boundary to lower temperatures, thus leaving the operating conditions of pipelines to be within a hydrate-safe region [1]. For the least, hydrates can cause blockages in pipelines, severely disrupting gas production, and also have the potential to cause explosions in pipelines. A common hydrate inhibitor that is utilized is Monoethylene glycol (MEG), it is mainly favorable due to its high recoverability. However, during the recoverability process MEG undergoes multiple phases of thermal exposure. This usually leads to thermal degradation in the MEG solution which results in an overall lower hydrate inhibitory performance [2].

In-order to understand how degradation occurs, its products, the impact on the equipment, and the hydrate inhibition performance of MEG, a method to degrade and test MEG is proposed in-detail. A study conducted by the authors that successfully utilized this method reported on the effect of regenerated MEG over multiple cycles [2]. The method essentially comprises of three stages; a) Degradation of MEG, b) Analysis of degraded MEG, and c) Hydrate testing of degraded MEG.

### Degradation of MEG

The utilization of MEG as a continuous hydrate inhibitor necessitates ongoing regeneration to remove impurities such as produced water, reservoir fluids, salts, corrosion products and production/drilling chemicals that have a tendency to accumulate within the MEG solution [[3], [4], [5], [6]]. Reclamation is the process in which non-volatile chemicals and monovalent salts are removed from the MEG solution through processing a stream of re-concentrated MEG solution from the regeneration process. The process occurs in a flash separator operating in vacuum where the input solution (MEG-water-contaminants) are boiled off at a temperature greater than the boiling point of water and MEG. Both, the water and MEG will evaporate while leaving behind salts and other chemicals that can then be removed from the system [7]. Care needs to be taken to ensure temperatures do not rise beyond the thermal degradation temperature of MEG, even though degradation of MEG has been shown to be possible at reclaimer operating conditions which are considerably lower [2,8].

**Fig. 1:**
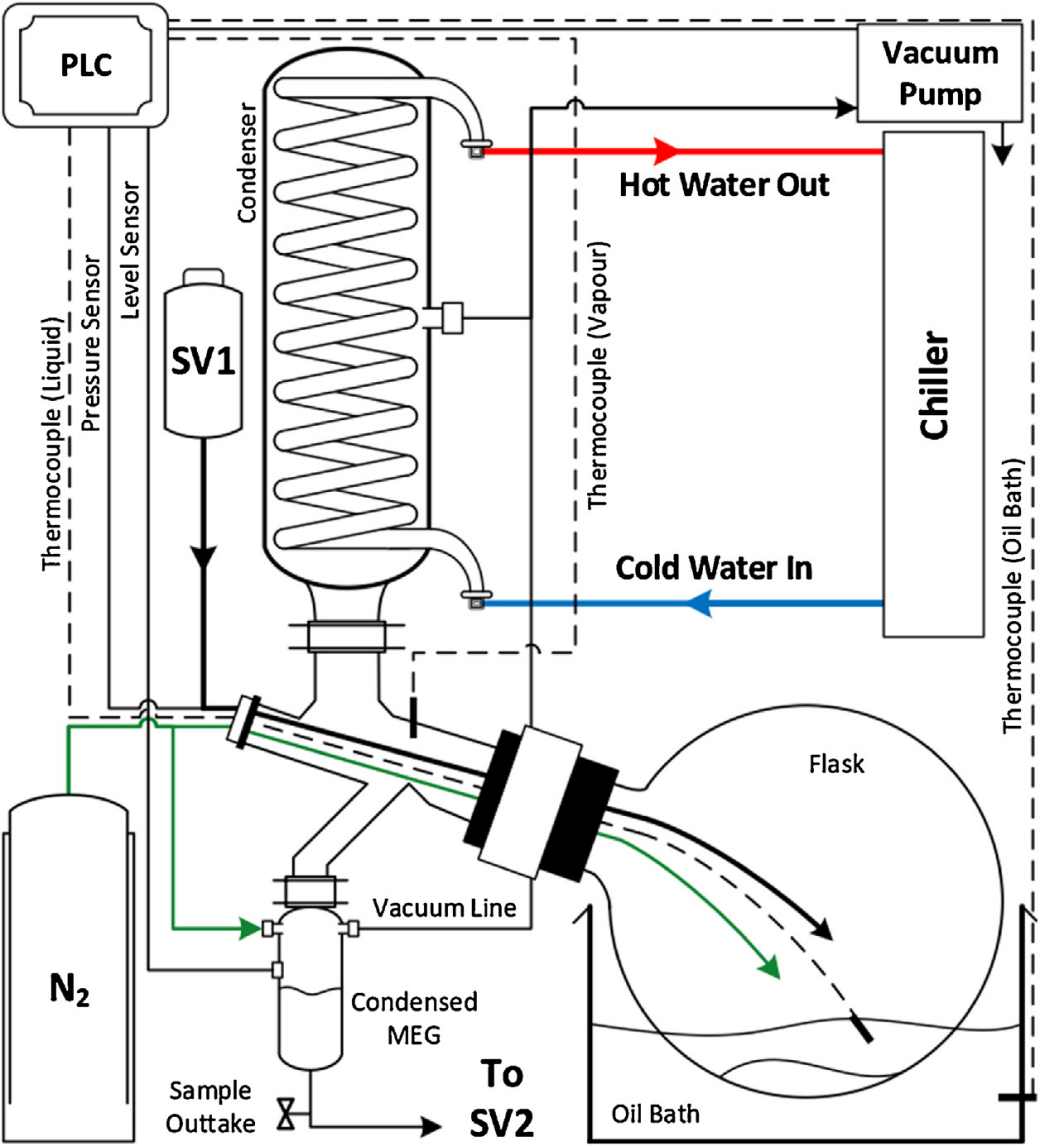
Schematic for the suggested experimental set-up of the reclamation unit.

Two experimental apparatuses within the laboratory (reclamation unit and autoclave system) will be illustrated and their procedures to produce degraded MEG samples will be outlined. The reclamation process typically implemented in the field was reproduced by a rotary evaporator essentially a vacuum distillation unit (Fig. 1). Laboratory scale rotary evaporators are designed for different reclamation processes with vacuum control with slight modifications based on specific requirements. The rotary evaporator is utilized to carry out the separation of MEG from monovalent salts and insoluble contaminants where salt-laden MEG as an input solution is distilled by removing the salts as a crystalized residue, and pure lean-MEG is collected as condensate product. To achieve optimum operating conditions, a vacuum pump is utilized to avoid MEG degradation due to high temperatures while increasing the salt removal efficiency. The reclamation unit comprises of an overhead condenser, a vacuum flask partially submerged in an oil bath, a vacuum system, a liquid receiver and an integrated control box. Modifications have been made to allow for sparging with nitrogen (99.999 mol%) to ensure there is no oxygen contamination. To ensure operating temperatures remain within tolerable and desired levels, several K-type thermocouples ware retrofitted to measure the temperatures of the vapor and liquid-slurry phases, while being connected to the Programmable Logical Controller (PLC). A level sensor was utilized to control the flow of lean MEG into the evaporator flask based on the desired slip stream portion from the input (or from the regeneration unit in the case of field application). Other instruments were utilized to monitor the system in terms of pH, pressure, flowrates, electrical conductivity (EC), dissolved oxygen (DO).

**Fig. 2:**
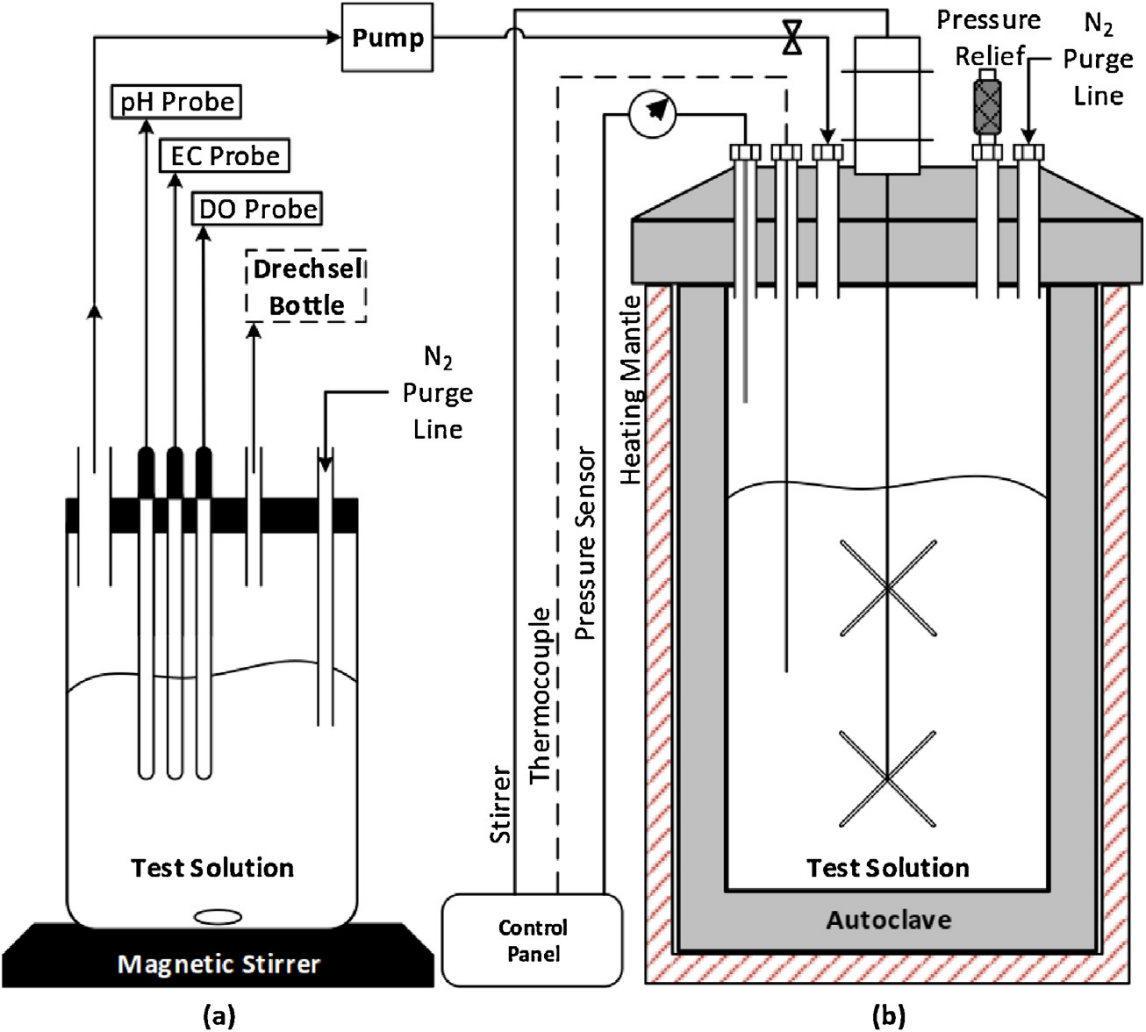
Schematic for the preparation of the test solution and autoclave set.
  (b) Prepare and transfer a salt-laden MEG solution according to desired concentration (typically MEG at 80 wt %) and volume based on experiment design into the beaker.
  (c) Give the solution sufficient time (6 h) for dissolved O_2_ levels to reach (≤20 ppb) and for complete synthesis.
2 Analysis of prepared solution representing non-degraded MEG.
  (a) Record all the measurements such as pH, EC, O_2_, color (photo) and mixing behavior.
  (b) Extract a smaller sample for IC to determine MEG degradation products (acetic, formic, glycolic acid).
  (c) Extract another sample in order to prepare a diluted MEG solution to a concentration of 20–40 wt % (typical field MEG injection concentration) for hydrate testing. Use Eqs. (1) and (2) to determine the required additional water (*ΔM*) to reach the desired MEG concentration for testing.
    (1) $$M_{1}C_{1}=M_{2}C_{2}$$
    (2) $$\Delta M=M_{2}-\;M_{1}$$
    where *M_1_* and *M_2_* are the masses of the initial (undiluted solution) and final (diluted solution) in g respectively, *C_1_* and *C_2_* are the concentrations of the initial and final solutions respectively, and *ΔM* is the additional water required to reach the desired concentration (*C_2_*) in g.
    After the careful preparation of the test solution, it is ready for the degradation process as follows:
3 Degradation of prepared solution using the reclamation unit (Fig. 1).
  (a) Transfer the initial solution to storage vessel 1 (SV1).
  (b) Power on the main PLC computer and in-line sensors such as pH, EC, DO, pressure and temperature.
  (c) Activate the nitrogen purge line to all vessels and the rotary flask to prevent unnecessary oxygen ingress.
  (d) Power on the cooling system and configure the temperature to around ∼4–6 °C.
  (e) Power on the liquid dosage pump from vessel 1 to start dosing into the rotary flask.
  (f) Power on the reclamation unit. The unit should be preconfigured to the desired refill, drain and condensate time as per experiment design.
  (g) Set-up the required vacuum pressure (10–15 kPa), oil bath temperature (depending on the required vapor temperature in the experiment design) and flask rotation speed (20–30 rpm).
  (h) Initiate the reclamation process and the flask shall start to receive salt-laden MEG at the preconfigured dosage pump flow-rate.
  (i) After sufficient drain and condensate time has occurred, the processed solution will be sent to storage vessel 2 (SV2).
  (j) MEG samples may be taken at any time from SV1/SV2 at the sample outtake valve for further analysis of degradation products and hydrate testing according to step 2.
  (k) When the volume level of SV1 is at 15%, activate the pump to transfer the contents of SV2 to SV1 so that the process can repeat until the total operation time for reclamation has been fulfilled according to the experiment design.
  (l) To shut-down the apparatus, drain the contents of the rotary flask and power off all equipment.
  (m) When sufficient cooling of the flask has occurred, extract the salt residue left at the bottom of the flask, and store it if required for future analysis (i.e. viscosity, SEM/ECM and particle analysis).
  (n) Extract the degraded MEG solution (contents of SV1 and SV2) for further analysis as outlined in step 2.
4 A slightly more simplified approach to attaining degraded MEG samples is the use of typical stainless steel high pressure/temperature autoclaves requiring no modifications (Fig. 2(b)). The procedure for MEG degradation using an autoclave is as follows:
  (a) Thoroughly clean the autoclave with ethanol and deionized water.
  (b) Transfer the prepared solution (step 1) to the autoclave using a pump to avoid unnecessary contamination of the autoclave.
  (c) Purge the autoclave for 2 h with nitrogen to ensure there is no oxygen contamination.
  (d) Place the autoclave in its heating jacket and activate the required temperature via the control panel.
  (e) Enable the pre-installed stirrer if required.
  (f) After the required operation time has passed, deactivate the system via the control panel and allow for the autoclave to cool down.
  (g) Once cool, extract the degraded MEG solution for further analysis as outlined in step 2.

Procedure for the preparation and degradation of test solution is as follows:

1 Preparation of initial solution (non-degraded salt-laden MEG solution)
  (a) Set-up the air-tight beaker system as shown in Fig. 2(a). The magnetic stirrer is used to mix and keep the solution in constant synthesis. Probes can be installed to measure pH, electrical conductivity and dissolved oxygen of the solution. Connect the nitrogen line to ensure there is minimum oxygen ingress.

### Hydrate testing of degraded MEG

**Fig. 3:**
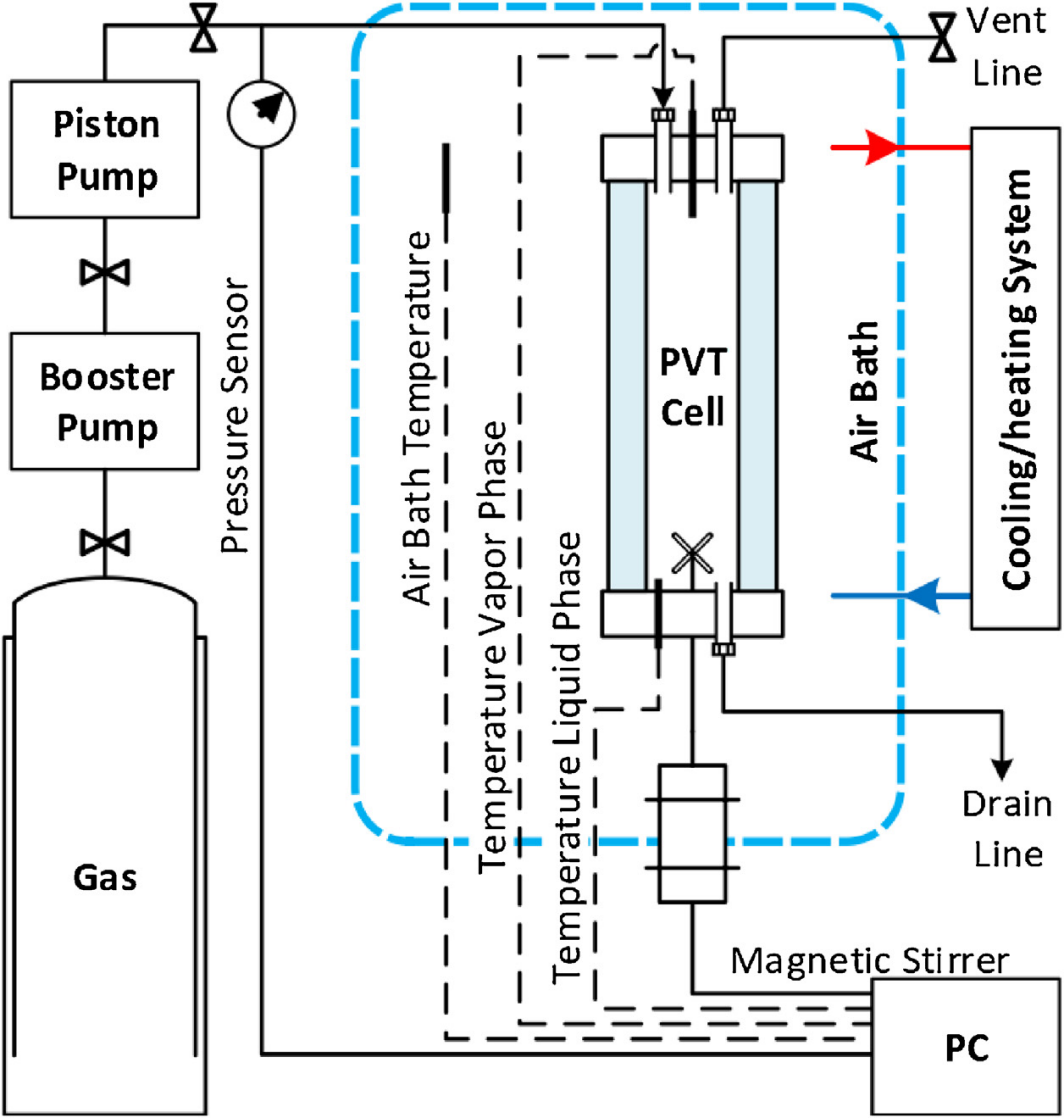
Schematic of experimental set-up for hydrate testing using PVT Cell.

To determine the hydrate phase equilibria of the degraded and non-degraded samples, a high pressure PVT Sapphire Cell can be utilized. The desired gas mixture can be introduced into the chamber according to the experimental design and the type of hydrate structure under study. Common methods of determining the hydrate phase equilibria can be employed such as the isochoric, isobaric and isothermal methods. A typical high-pressure PVT cell (Fig. 3) is made out of sapphire material so a complete visual of the internals of the chamber is available for detailed visual observations. The cell has been designed with an inner volume of 60 cm^3^ to allow for sufficient gas and liquid to form hydrate. An automated magnetic stirrer fitted to the cell produced an agitation rate that helps in the complete transformation of the liquid water phase to hydrate, and encourages the renewal of the surface where there is a higher tendency for hydrate film to form. The recommended stirrer rate to be applied is 400–500 rpm. The cell is equipped with pressure and temperature sensors to capture PVT data for further analysis.

5 Method to determine the phase equilibria for degraded MEG solution
  (a) Thoroughly rinse the inside of the PVT cell with ethanol/acetone, and then with deionized water.
  (b) Close all valves and power on the vacuum pump to ensure there are no contaminants within the cell.
  (c) Inject a 7 mL sample of the test solution through the inlet valve into the cell.
  (d) Power on the PVT system (control computer, piston pump, magnetic stirrer, air circulation fan and cooling system).
  (e) Ensure the gas supply is ready and firmly connected to the manifold then open the gas input line into the cell.
  (f) Enable the piston pump via the control software to inject gas into the chamber and to increase the pressure to the desired pressure for the first point on the hydrate phase boundary. Close the gas input valve once desired pressure is achieved.
  (g) Enable the heating system to heat up the sample to 35 °C to destroy any water memory profiles, then turn off the heater.
  (h) Enable data acquisition and ensure temperature, pressure and stirrer rate data are being recorded (at 5 s intervals).
  (i) Begin video recording using the camera and light beam focused on the sample within the cell.
  (j) Enable the cooling process to begin and set the cooling rate to 1 °C/h via the control software.
  (k) Carefully note visual observations such as the growth, agglomeration and behavior of hydrate formation; the inter-phase conditions (i.e., clear, foaming, bubbling, grey or cloudy), film formation on the inner walls of the cell; the temperature at which the ﬁrst hydrate particle is formed, the point at which the stirrer stops moving due to impeding hydrate solids, and the rate of reduction of the solution in the cell.
  (l) When all visible liquid has transformed into hydrate, continue the cooling process for a further 3 °C but avoid going below 0 °C (i.e., ice formation region).
  (m) Begin the slow step-wise heating process at a rate of 0.5 °C/h with a maximum rate of 1 °C/h so that a sufficient time is available for equilibrium to be achieved. The process can be ended when all visible hydrate solids are converted to liquid.
  (n) The PVT system can now be cleaned and shut-down.
  (o) From the acquired temperature and pressure data for the cooling and heating processes, the hydrate thermodynamic equilibrium point may then be determined from the intercept of the two curves. Use the computer script provided in the Supporting information for automated processing of data logs to determine the hydrate phase equilibria conditions.
  (p) Repeat the entire process (5) for at least another 3 more pressure points in order to plot the hydrate phase boundary.

### Method validation

**Fig. 4:**
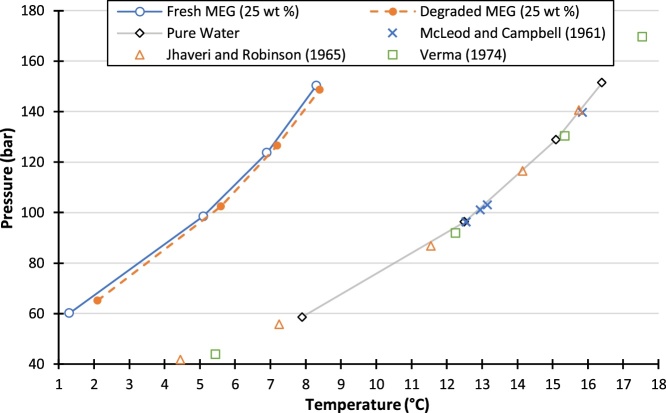
Comparison of degraded MEG with fresh MEG, and literature comparison of methane-water hydrate.

The degradation of MEG can be identified by the presence of by-products. Studies from literature that investigated degradation of MEG have found the by-products of MEG degradation to be formic, glycolic, acetic and oxalic [[9], [10], [11], [12], [13], [14]]. Numerous studies have been conducted by our laboratory using our method which are outlined in Table 1 [2,15,16]. The results clearly show the presence of degradation products such as acetic acid between degraded and non-degraded samples. A study conducted by Psarrou et al., (2011) has reported that a sign of degradation in the reclamation process is the color of the solution where it changes to more of a yellow color [8]. The color changes have also been reported in Table 1, and it can be seen that the color has changed from clear to yellow to dark brown as the degradation amount increases amongst the MEG solution samples. Furthermore, the effect of MEG degradation on the hydrate phase boundary can be studied using this method. A pure MEG solution of 25 wt % was prepared and degraded for 100 h using this method. The changes in color, pH, EC and the shift in hydrate phase boundary have been reported in Table 1 and Fig. 4. It can be confirmed that degradation products and promotion of hydrate formation was found.

**Table 1:** Experimental data of degraded and non-degraded MEG solutions using reported methods.

Solution	Exposure Temperature (° C)	Exposure Time (h)	ΔT_Hyd_ (° C)^a^	Color	ΔpH a	ΔEC (μS/cm) a	Acetic acid (ppm)	Source
MEG (25 wt %)	23.6^b^	-b	0		0	0	3	-c
	100	100	0.18		−0.15	43	10	
MEG (25 wt %)	135	48	–		–	–	12	[15]
	165	48	0.72		–	–	18	
	185	48	1.07		–	–	21	
	200	48	1.62	–	–	–	–	
MEG (20 wt %) + MDEA (2 wt %)	22^b^	-b	0		0	2	10	[16]
	135	240	1.7		−0.29	50.1	36	
	165	240	1		−0.43	78.9	56	
	185	240	1.1		−0.45	112.0	62	
	200	240	1.3		−0.56	141.3	71	
MEG (20 wt %) + Brine	≤126	11	0.13		–	–	6.5	[2]
	≤126	56	1		–	–	82.7	
	≤126	97	1.7		–	–	139.3	

a Shift from a non-degraded sample of the same solution.

b Room conditions.

c This study.

Experiments were conducted to determine the methane-water hydrate phase boundary using the set-up reported in this study. The phase equilibria data are plotted in Fig. 4. When compared to the widely available literature data [[17], [18], [19]], an absolute average relative error (AARE) of 0.98% was found, which confirms that our apparatus and procedure are highly accurate in determining hydrate phase equilibria (Fig. 4).

## Conclusion

Flow assurance challenges such as gas hydrates and corrosion are a serious concern for the oil and gas industry. An array of chemicals (i.e., hydrate, corrosion, scale, wax inhibitors and oxygen scavengers) are injected into the hydrocarbon production and process pipelines to prevent, decrease and or mitigate these concerns. MEG is a conventional hydrate inhibitor that is commonly used in the industry due to its reusability. However, MEG may undergo degradation in the reboiler and reclamation units of a MEG regeneration plant. Thus, to study the effects of degradation of MEG especially in the presence of other chemical additives upon the adopted hydrate inhibition program becomes important. This study has outlined the necessary methods to mimic field-like degradation of MEG and analysis in terms of hydrate inhibition performance and degradation products.
