# Supplementary material for: Degradation and hydrate phase equilibria measurement methods of monoethylene glycol
Source: MethodsX. 2018 Dec 4;6:6–14. doi: 10.1016/j.mex.2018.12.004 (PMC6308260; doi:10.1016/j.mex.2018.12.004)
Supplement: Supplementary file 1 [file mmc1.docx]

**SUPPLEMENTARY MATERIAL**

Computer script to process raw data and determine the hydrate phase equilibrium conditions.

function P = HydEqm(HF,filename)

% Function HydEqm finds and plots the hydrate phase equilibrium point

% from raw PT data.

% The function requires the observed hydrate formation point to remove % unnecessary data and the source pressure/temperature data from the

% cooling/heating process using the isochoric test method.

%

% Author : Khalid Alef

% Version: 1.0, 31 July. 2018

rawdata = csvread(filename,2,1); *%..read and import raw PT data*

data = rawdata(:,1:2); *%..remove unnecessary data*

data(:,1) = data(:,1)./100; *%..convert data to desired units*

plot(data(:,2),data(:,1))

idx = data(:,2) < HF; *%..remove unnecessary data*

modiData = data;

modiData(idx,:)=[];

di = modiData(2:end,1)-modiData(1:end-1,1);

cutoff = find(di==max(di));

line1 = modiData(1:cutoff,:); *%..separate cooling and heating*

line2 = modiData(cutoff:end,:);

pt1 = polyfit(line1(:,2),line1(:,1),1); *%..fit linear trends*

pt2 = polyfit(line2(:,2),line2(:,1),1);

x_intsect = fzero(@(x) polyval(pt1-pt2,x),3); *%..intersection*

y_intsect = polyval(pt1,x_intsect);

P(1)=x_intsect;

P(2)=y_intsect;

range = HF:0.001:max(modiData(:,2));

val1 = polyval(pt1,range);

val2 = polyval(pt2,range);

figure *%..plot the data and trend-lines*

plot(line1(:,2),line1(:,1),'co',line2(:,2),line2(:,1),'mo')

hold on

scatter(P(1),P(2),'filled')

plot(range,val1,'b',range,val2,'r')

output = P; *%..hydrate equilibrium pressure and temperature*

end
